# The Surgical Treatment of Stone

**Published:** 1912-01-27

**Authors:** John Ward Cousins

**Affiliations:** Consulting Surgeon to the Royal Portsmouth Hospital.


### THE SURGICAL TREATMENT OF STONE.

### A Review of its Practice during the Last Sixty Years.—II.*

By JOHN WARD COUSINS, M.D., F.R.C.S., Consulting Surgeon to the Royal Portsmouth Hospital.

### Lithotrity.

During the first ten years of my practice I was present at only a few cases of lithotrity, for the method of crushing stone at that time was still in an early stage of evolution. The operation as then performed had many drawbacks, and one crushing was seldom sufficient to secure a complete cure. Fragments of stone were often left behind in the bladder and caused much irritation, and sometimes serious inflammatory changes, by blocking the urethra in their outward passage. Many operators advocated short sittings without the administration of either chloroform or ether for the purpose of avoiding any injury to the bladder during their manipulations. Experience soon overcame these prejudices and the use of anæsthetics was followed by considerable progress.

About this period Sir Henry Thompson successfully modified many details, and improved the old crushing instrument by adapting to it a special screw arrangement. During the early seventies lithotrity was generally recognised as the best operation for many cases of stone, and it steadily gained in reputation. The practice of carefully washing out the bladder after each sitting was now very generally adopted, and a scoop too was used for bringing away the finer detritus. Some surgeons preferred a double catheter which permitted the injection of a large quantity of water. In several cases I employed Clovers' simple and ingenious instrument for the removal of the last fragments. All these improvements of the operation of lithotrity, however, did not completely supersede the practice of lithotomy, but surgeons generally were calmly and impartially seeking the best method for the relief of the cases which fell into their hands.

### Introduction of Litholapaxy.

In 1878 Professor Bigelow, the distinguished surgeon of Boston, introduced the proceeding now universally known as litholapaxy, by which he succeeded, after breaking up the stone with a powerful lithotrite, in evacuating at once all the triturated fragments. This new method rapidly took the place of the old crushing operation, and it was accepted as a great advance in the surgical treatment of vesical calculus by which the mortality could be reduced and the suffering connected with the disease greatly mitigated. Bigelow was the first surgeon to utilise Dr. Otis's investigations respecting the size of the male urethra. He proved the full dilatibility of the canal, and the possibility of using a full-sized evacuating tube for the removal by aspiration of every fragment of a crushed calculus at one sitting. This was the special advantage of the operation, and the immediate results were both brilliant and successful, and at the same time the risk of recurrent formations was greatly reduced.

At the onset in this country litholapaxy was regarded as only suitable for adult patients, but recently the practice of Dr. Beaumont, Dr. Keegan, and other surgeons in India have clearly proved that it can be successfully employed in the treatment of stone even in young children. The urethra of boys from four to six years of age will bear the careful introduction of a small No. 7 or No. 8 lithotrite and a No. 8 evacuating catheter, and with these instruments many small calculi have been broken up and removed. I have done many operations with the greatly improved instruments of Bigelow and Thompson, but I have never performed a crushing manipulation on a young male child; at the same time I feel confident that the new method is well adapted in many cases for the removal of small stones in children, and that it has many advantages over supra-pubic and lateral lithotomy, and can be undertaken with greatly reduced danger.

Litholapaxy in the aged is often a complicated proceeding from the presence of chronic changes in the bladder and prostatic obstruction which render it both risky and difficult. It is my experience that this operation should not be undertaken in any case complicated with septic disturbance in any part of the urinary track until marked improvement has been established by local treatment and drainage. In two cases of this description I opened the bladder freely through the perineum to establish efficient drainage. I then crushed the stone through the wound with long and powerful forceps similar to the instrument used by Reginald Harrison, and washed away all fragments with the aid of a long evacuator. One of these patients was a man of fifty years of age suffering from stone and many perineal sinuses, the result of long-standing stricture of the urethra. The operation took place fifteen years ago, and recently I heard that he was in good health and quite free from bladder trouble. The other patient was a young man who had been suffering for a long period from calculus and fœtid cystitis. The operation gave him great relief and he went home at the end of two months, but I heard that he died from renal disease some time after his return.

### Revival of Supra-pubic Lithotomy.

I must now refer to the great change which has taken place during the last few years by the revival of supra-pubic lithotomy. During the early period of my professional life I never had an opportunity of seeing this operation performed, as the perineal methods were almost universally practised, and the removal of a stone by an incision through the abdominal wall, and then extending it underneath the reflection of the peritoneum into the bladder, had gone out of fashion for many years. I can fairly state, however, that I nearly on one occasion had an opportunity of doing it. In the year 1873 an old man of seventy was sent from the country to the Royal Portsmouth Hospital in a state of great depression and suffering from retention of urine. He

* The first articles appeared last week, p. 405.

had been troubled for a long period from incontinence and constant pain, but the presence of stone had never been detected. On exploring the bladder the whole cavity was found to be distended with a hard concretion, and I at once decided to do the high operation, provided the patient rallied and his general condition permitted me to interfere. He suddenly died early the next day, and at the post-mortem examination I removed through the abdominal wall a hard stone of twelve ounces in weight, which was completely moulded to the inside of the cavity. Both ureters were greatly thickened and dilated, and each of them contained several ounces of urine. The specimen is now in the Rundle Museum of the hospital, and here are also to be seen a large number of the calculi I have removed by operation.

### The Revival of the Eighties.

Before the remarkable revival which took place about five-and-twenty years ago the old surgical records of supra-pubic lithotomy exhibited very unfavourable results, and clearly indicated that its practice by operators in every country had been attended with a very high mortality. Surgeons were long divided in their opinions as to the difficulties and disadvantages of the method until these were finally dissipated by the advance of abdominal surgery, and the rapid progress of antiseptic treatment. By the efforts, too, of Garson in Edinburgh, Christopher Heath and Henry Thompson in London, the technique of the operation was improved and their successful practice promoted all round the examination and revival. At this time many details were introduced which were intended to render the procedure both easy and safe. Some surgeons recommended the use of a hollow sound fitted with a stylette for injecting the bladder, and also as a guide in making the deep incision. The distension of the rectum with a rubber bag was also considered a valuable help for the purpose of raising the floor of the bladder as much as possible within reach of the finger. The bag was a pear-shaped contrivance of soft rubber with a tube attached to it, and after introduction it was carefully filled with warm water; but recent experience has proved that both the urethral guide and the rectal bag—except perhaps in very special cases—possess very little utility, and now they are seldom employed. I have often operated with no other dilator of the rectum than the finger of an assistant protected by a rubber glove. The peritoneal fold varies of course with the age of the patient and the amount of fluid contained in the bladder, but with ordinary precaution and the sparing use of the knife there is really no risk of an accident. Some years ago I was invited by a very able surgeon to be present at a supra-pubic operation, and unfortunately instead of opening the bladder he cut into the intestine by mistake.

Now, one word with reference to the position of supra-pubic lithotomy. To-day—modified by the results of practice and every up-to-date surgical precaution—I feel confident that it will be the operation of selection in the future; and, although it cannot be performed with the rapidity and brilliancy of the old lateral method, it is a proceeding of far greater safety, and is especially suitable for all young children, for all cases of large and hard calculi, and also in cases in which the urethra from any cause will not admit the passage of a lithotrite.

### Stone in Women.

Stone in women is not a very common disorder, and occurs much less frequently than in men, and this is in a great measure due to the short and capacious female urethra which permits small concretions to escape during the early stage of their formation. Some years ago I was consulted by a patient suffering from a chronic urinary trouble, and she brought with her over fifty small round calculi which she had passed during the morning. In the large majority of cases the removal of a stone from the female bladder is not a difficult proceeding, and many cases can be successfully treated after simple dilatation of the urethra. My first operation for stone was on a girl twelve years of age. It was a small flat concretion nearly an inch in its broadest diameter, and I found it to be too large for safe extraction after dilatation, so I passed a director along the urethra, and then with a narrow probe-pointed knife very sparingly notched the passage at the neck of the bladder. I was greatly disappointed with the result, for she left the hospital suffering from incontinence of urine, and this continued more or less for two years, and then I lost sight of her. After twenty years she unexpectedly called upon me at Southsea, and then I heard from her with great satisfaction that she had completely recovered from her old trouble, had been married for many years, and was the mother of several healthy children.

### Vaginal Lithotomy.

In some cases of stone in women crushing can be practised with success, and the fragments removed through an evacuator. Some years ago I had a short lithotrite made about eleven inches in length and the size of a No. 10 catheter; but the difficulty of keeping the bladder sufficiently full for the manipulations rendered the operation long and tedious. For children with a hard calculus which could not be well removed by dilatation I have used sometimes another simple contrivance, and this consists in a pair of forceps with short fenestrated blades fitted with long handles with a screw for crushing the stone. The joint of the forceps is surrounded with a conical bag made of thin rubber, which can be inflated to plug the urethra. After the introduction of the instrument water is injected into the bladder through a small tube which is fixed in the rubber plug. By just raising the child the stone can be made to fall between the blades of the instrument. In cases of large stone at all ages the supra-pubic operation is in my opinion far preferable to vaginal lithotomy. I have never done the latter operation, but I have had to close a large vesico-vaginal fistula which was the result of this procedure.

*(To be continued.)*

